# Coverage, quality of and barriers to postnatal care in rural Hebei, China: a mixed method study

**DOI:** 10.1186/1471-2393-14-31

**Published:** 2014-01-18

**Authors:** Li Chen, Wu Qiong, Michelle Helena van Velthoven, Zhang Yanfeng, Zhang Shuyi, Li Ye, Wang Wei, Du Xiaozhen, Zhang Ting

**Affiliations:** 1 Department of Integrated Early Childhood Development, Capital Institute of Paediatrics, Beijing, China; 2 Global eHealth Unit, Department of Primary Care and Public Health, Imperial College London, London, UK; 3 Department of Molecular Immunology, Capital Institute of Paediatrics, Beijing, China

**Keywords:** Postnatal care, Coverage, Quality, Mixed method study

## Abstract

**Background:**

Postnatal care is an important link in the continuum of care for maternal and child health. However, coverage and quality of postnatal care are poor in low- and middle-income countries. In 2009, the Chinese government set a policy providing free postnatal care services to all mothers and their newborns in China. Our study aimed at exploring coverage, quality of care, reasons for not receiving and barriers to providing postnatal care after introduction of this new policy.

**Methods:**

We carried out a mixed method study in Zhao County, Hebei Province, China from July to August 2011. To quantify the coverage, quality of care and reasons for not using postnatal care, we conducted a household survey with 1601 caregivers of children younger than two years of age. We also conducted semi-structured interviews with 24 township maternal and child healthcare workers to evaluate their views on workload, in-service training and barriers to postnatal home visits.

**Results:**

Of 1442 (90% of surveyed caregivers) women who completed the postnatal care survey module, 8% received a timely postnatal home visit (within one week after delivery) and 24% of women received postnatal care within 42 days after delivery. Among women who received postnatal care, 37% received counseling or guidance on infant feeding and 32% on cord care. 24% of women reported that the service provider checked jaundice of their newborns and 18% were consulted on danger signs and thermal care of their newborns. Of 991 mothers who did not seek postnatal care within 42 days after birth, 65% of them said that they did not knew about postnatal care and 24% of them thought it was unnecessary. Qualitative findings revealed that staff shortages and inconvenient transportation limited maternal and child healthcare workers in reaching out to women at home. In addition, maternal and child healthcare workers said that in-service training was inadequate and more training on postnatal care, hands-on practice, and supervision were needed.

**Conclusions:**

Coverage and quality of postnatal care were low in rural Hebei Province and far below the targets set by Chinese government. We identified barriers both from the supply and demand side.

## Background

The postnatal period is defined by the World Health Organization (WHO) as the period from one hour after the delivery of the placenta to six weeks after birth [1]. The postnatal period is a critical transitional time for a woman and her newborn physiologically, emotionally, and socially [2].

Every year, 2.9 million babies die in their first four weeks of life (the neonatal period) globally [3]. While mortality of children has dramatically dropped in the past decades, there has been less progress in decreasing neonatal mortality. Between 1980 and 2000, there was a 25% reduction in the neonatal mortality rate. This was smaller compared to the 33% reduction in the mortality rate of children aged two months to five years [3,4]. China has achieved a remarkable decrease in the neonatal mortality rate (62% reduction from 1996–2008) with an annual decline rate of 8.3% [5,6]. However, given the large population in China, still an estimated 416,000 newborns died and this accounted for 10% of global neonatal deaths in 2000 [5].

The maternal mortality in China also declined steadily from 80 per 100,000 live births in 1991 to 26.1 per 100,000 live births in 2011 [7]. However, many women suffered from more than one physical disorder after childbirth such as stress incontinence, perineal pain, breast problems, backache, haemorrhoids and constipation worldwide as well as in China [8-10]. Postnatal depression, one of the most frequent psychological disorders, also affects the lives and well-being of women and their families. The reported prevalences of postnatal depression are about 10-15% worldwide [11] and on average 14.7% in China [12]. Most studies reported that many women did not report their physical or psychological problems to health professionals or even to their family members, which indicates that more and improved care is needed during the postnatal period [11].

Family, community, outreach and facility-based care are the essential postnatal health service delivery modes. Postnatal care has proved to be more cost-effective in decreasing neonatal mortality than antenatal care and intrapartum care [13]. Postnatal care interventions that focused on the identification and management of women’s health problems also have shown to improve physiological and psychological health [11,14,15]. Effective and cost-effective neonatal survival interventions have been identified and consensus has been reached to scale-up these interventions in health systems in low- and middle- income countries [13].

However, the coverage of postnatal care in low- and middle-income countries is low [16-18]. WHO and the United Nations Children’s Fund (UNICEF) guidelines recommend a postnatal care visit for the mother and her newborn on day 1, day 3, and day 7 after birth, with continuing contact throughout the first six weeks of life [19]. In 1989, the Ministry of Health of China set the targets for at least three postnatal care visits for both women and children to be 70% in urban and 50% in rural areas [20]. Still in 2008, the coverage of at least one postnatal care visit was 61% in urban and 54% in rural areas according to the latest National Health Services Survey (NHSS) [21]. In addition, some evidence suggests that postnatal homecare rates were even lower. A study in Anhui Province, China found that only around 4% of women received at least one postnatal home visit [22]. Care for mothers and their newborns after birth has received little emphasis in both public health policies and programs globally and in China [21,23]. Antenatal and intrapartum care have received much more attention, which is reflected in high coverage rates for antenatal care and hospital delivery (over 90%) and considerably lower postnatal care visit coverage (56%) across China [21].

In China, the Maternal and Child Health Services Division at National Health and Family Planning Commission provides overall direction for maternal and child health services [24]. Ministerial directives are translated by the provincial departments into implementation plans at city, county, township, and village levels [25]. In rural areas, a three-tier maternal and child healthcare system that covers county, township and village levels was established back in the 1950s. Now at county level, there is a maternal and child health centre and health workers there provide antenatal and postnatal care and undertake surveillance of infants, preschoolers and school children. County health workers are also responsible for the training and supervision of township and lower level health workers [25].Township health workers, who receive secondary school medical training (after 9 years of basic education) or junior college (after 12 years of basic education) in western or Chinese medicine, are the main provider of antenatal care, postnatal care and immunization in their catchment areas [26,27]. Village clinics are at the bottom tier where village doctors provide both curative and preventive health services [27,28]. Village doctors predominantly attend technical school and receive training in western medicine. While township health workers and village doctors are not specifically trained to conduct maternal and child health care services, they are responsible to perform postnatal care in rural China [29].

In 2009, Chinese government launched free antenatal care and postnatal care services for all urban and rural residents as part of the nine basic public health services [30,31]. National policy and guidelines have emphasized that at least one postnatal home visit for women and children within one week after delivery, followed by a facility healthcare visit for women and children within 42 days after delivery is needed (Additional file 1). As part of the medical reform plan, the Chinese government has invested in improving the infrastructure of primary health facilities, such as township hospitals and village clinics [32]. Also in-service training of health workers on antenatal care and postnatal care has been conducted [33].

However, there are still several known barriers for women to receive postnatal care. In Chinese culture, traditional “zuoyuezi”, literally meaning “sitting the first month after delivery” (or “sitting month” in brief) restricts women from going out of their home or receiving visits from others [22,34,35]. Therefore, healthcare demand of women during the postnatal period is not as strong as during the antenatal and intrapartum periods. Before the initiation of the free postnatal care services, one study reported that a lack of maternal and child healthcare (MCH) workers and inadequate training on postnatal care contributed to the suboptimal coverage of postnatal care services [35]. However, no studies have looked into women’s reasons for not receiving and healthcare workers’ barriers to provide postnatal care after initiation of the free postnatal health services.

This study aimed to explore coverage, quality of care and barriers to postnatal care, in rural Hebei, China, after introduction of the free postnatal care services by the Chinese government in 2009. We aimed to not only evaluate how postnatal care was conducted, but also to increase our understanding of why women do not receive postnatal care and why healthcare workers do not provide care. Therefore, we used mixed methods that include a quantitative survey with women and qualitative semi-structured interviews with MCH workers.

## Methods

The data in this paper were generated as part of a broader research project on maternal and child healthcare services in rural China, Zhao County in Hebei Province, entitled ‘Effectiveness of a scaling-up model for child health interventions: a cluster randomized control trial’ (unpublished).

### Definition of postnatal care

In our study, two different types of postnatal care were included: 1) postnatal home visits and 3) postnatal care in a healthcare facility within 42 days after delivery. The coverage of postnatal home visits was defined as the percentage of women and/or children who were visited at home during the postnatal period. Coverage of postnatal care in a healthcare facility within 42 days after delivery was defined as the percentage of women and/or children who received postnatal services at any healthcare facility within 42 days after delivery. For these indicators, the denominator was surveyed women who had a child younger than two years. Quality of postnatal home visits in our study was assessed in terms of the Newborn Indicators Technical Working Group recommended services [19] that neonates received: checking the newborn’s umbilical cord; assessing the newborn’s temperature; observation of and counseling for breastfeeding and counseling on newborn danger signs and weighing the baby. In addition, we added one other service: checking the jaundice.

### Study area

Hebei province is located in the northern part of North China Plain, with an area of 190,000 square kilometers, bordering Beijing. At the end of 2008, the total population in Hebei province was 69,890,000, of which 58% lived in rural areas. Hebei has 11 prefectures, 172 counties, 2,228 townships and 49,216 villages. The net income per capita of rural residents in 2010 was 5958 Yuan (equal to 956 USD) [36], nearly the same as the national level (5919 Yuan, equal to 950 USD) [37]. Zhao County is located 40 kilometers south from Shijiazhuang, the capital city of Hebei province, with an area of 675 square kilometers. In 2010, Zhao County had a total population of around 571,000 and the net income per capita of rural residents was 6464 Yuan (equal to 1038 USD) (data from Zhao county statistics bureau, unpublished). There are 344 health facilities in Zhao County, with three county-level public hospitals, 16 township hospitals, 281 village clinics and four private hospitals.

### Mixed methods

We used mixed methods and combined a quantitative household survey and qualitative semi-structured interviews. Mixed methods research can be viewed as an approach which draws upon perspectives of quantitative and qualitative research methods. This is increasingly recognized as valuable, because it can potentially capitalize on the respective strengths of quantitative and qualitative approaches [38]. Generally, the purpose of combining qualitative research and quantitative research can be: 1) triangulation, that is to validate different sources of data; 2) complementary, that is to clarify, explain or more fully elaborate results; 3) development, that is to guide further sampling, data collection and analysis [39]. In this study, we used qualitative research for the second purpose, with the aim of increasing our understanding on barriers for healthcare workers to provide postnatal care.

### Quantitative study

We conducted a Maternal, Newborn and Child Health Household Survey (MNCH HHS) with 1601 caregivers of children younger than two years of age to quantify coverage, quality of care and the reasons for not using postnatal care. The MNCH HHS was conducted from 15 to 24 August 2011. MNCH HHS was developed by WHO and then translated into Chinese and adapted to the Chinese context in 2010 [26]. The sample size and sampling method for the survey was based on the cluster randomized control trial in Zhao County and the survey was used as the baseline assessment for the trial (Additional file 2). The inclusion criterion of our study was children who were registered as permanent residents. Exclusion criteria were children who were not permanent residents and those who were permanent residents, but currently did not live in the particular village on the name list. Trained interviewers used smartphones with pre-stored survey software to acquire information on general family characteristics, antenatal care, delivery and postnatal care, infant and young child feeding, immunization and child healthcare [40].

### Qualitative study

We developed semi-structured questionnaires to conduct interviews with MCH workers at township level (Additional file 3), since MCH workers were assigned by the government to provide postnatal care visits [31]. We assumed that MCH workers could better inform us of barriers to provide postnatal care visits than caregivers, because postnatal home visit require MCH workers to actively reach out to families in their catchment areas. We found in our household survey in 2010 (unpublished data) that coverage of home visits was lower compared to health facility visits within 42 days, and therefore we only focused on exploring barriers to postnatal home visits. We developed topic guides based on our findings from a previous household survey (2010) [26] and in discussion with local MCH hospital staff. The purpose of the interviews was to explore perceptions of MCH workers on general workload, income, training and postnatal home visits. After training, four researchers from Capital Institute of Pediatrics and provincial-level MCH hospitals conducted semi-structured interviews from 15 to 24 July 2011. We aimed to interview 32 health workers: one maternal healthcare worker and one child healthcare worker from each of the 16 townships. However, we only conducted 24 interviews, because in 7 townships the MCH workers conducted both the maternal and child healthcare work and one MCH worker refused to participate. Interviews with MCH workers were done at their workplaces, which was convenient for them. Interviews were conducted in Mandarin, typically lasted around 30 minutes, and were digitally recorded with the permission of each participant. All interviews and discussions were then transcribed verbatim in Chinese by two independent investigators. A third investigator checked the consistency of the transcripts and verified the transcripts by listening to the tapes again.

### Data management and analysis

We uploaded MNCH HHS data onto an internet server and data were automatically transformed into a Microsoft Excel sheet. After data cleaning, we converted the database into a database file for final analysis. We used the median and interquartile range (IQR, Q1-Q3) to describe the central tendency and dispersion of continuous variables and we used proportions to describe binary or categorical variables. Two-sample wilcoxon rank-sum test was used to compare continuous variables between women who did and did not seek care within 42 days after delivery. Pearson chi-square test and Fisher exact test were used to compare binary or categorical variables between women who did and did not seek care within 42 days after delivery. Statistics were performed using Stata Statistical Software: Release 11 (College Station, TX: StataCorp LP). We first did the analysis of quantitative data to assess the coverage and quality of postnatal care. Then we analyzed the qualitative research data to further explore barriers that MCH workers experienced when visiting women during the postnatal period.

Thematic framework analysis [41] was used to classify and organize the semi-structured interviews according to key themes. Two researchers (CL and WQ) first independently read the transcripts to identify key themes. Then CL and WQ coded quotes, listed quotes related to our research question and organized the quotes into key themes in a table (in Mandarin). We used the tables to describe behaviors, beliefs and reasons that were similar or different, and to develop explanations and explore associations. The two researchers discussed areas of agreement and discrepancies and further refined the coding scheme until consensus was reached on the findings and on the explanation from the analysis. Finally, CL translated the themes and related quotes into English and WQ reviewed the translated themes. We list all the key themes that we identified and related illustrative quotes.

### Ethical approval and informed consent

The Ethical Committee of Capital Institute of Pediatrics approved the study. All health workers and caregivers involved into this study read the informed consent form and gave their written consent.

## Results

### General characteristics of participants

A total of 1601 caregivers participated in our survey. The median age of mothers was 27 years old and most of them (99%) had a rural Hukou (residence) (Table 1). Nearly 80% of mothers completed junior high school and 75% were farmers. The median annual family income was RMB 20,000 Yuan (equal to USD 3,200) and the median annual family consumption expenditure was RMB 15,000 Yuan (equal to USD 2,400). The gender ratio of children was 134:100 (boys to girls), indicating that there were more boys than girls in our survey.

**Table 1:** **Characteristics of participants and their family in rural Hebei, China 2011 (N = 1601)**^**$**^

**Characteristics**	**n**	**%**
Maternal age, yr*	27	(24–30)
Maternal Hukou		
Urban	20	1.26
Rural	1563	98.74
Maternal education		
Primary school	82	5.27
Junior high school	1238	79.51
High school	187	12.01
College or above	50	3.21
Maternal occupation		
Farmer	1186	74.92
Worker/Staff	64	4.04
Housewife	278	17.56
Others	55	3.47
Family size*	5	(4–6)
Annual family income, Yuan*	20000	(10000–30000)
Annual family consumption* expenditure, Yuan*	15000	(10000–20000)
Children’s gender		
Male	916	57.21
Female	685	42.79

^$^18 were missing for maternal age, maternal Hukou and maternal occupation respectively, 44 were missing for maternal education, 638 were missing for annual family income and 603 were missing for annual family consumption expenditure.

*Continuous variables were expressed as median (Q1-Q3).

We conducted 24 semi-structure interviews; two interviews were not included into our final analysis, because the interviewed MCH workers left in the middle of the interview as they had to go back to work. Among 22 MCH workers who completed the interview, 18 (82%) of them graduated from a secondary technical school (three years of professional study after junior high school) (Table 2). Eight MCH workers had a major in western medicine and four had a major in Chinese and western medicine. Eight MCH workers had been working for more than 10 years and nine had been working for less than five years. Seven MCH health workers undertook both the maternal and child healthcare work. We identified three major themes: 1) providers of postnatal home care; 2) services provided during postnatal home visits; 3) barriers to postnatal home visits. Each theme is presented with the quantitative findings below.

**Table 2:** Characteristics of the interviewed maternal and child healthcare workers (N = 22)

**General characteristics**	**n**	**%**
Medical education		
Secondary technical school	18	81.82
College level	4	18.18
Major		
Chinese and Western Medicine	4	18.18
Western Medicine	8	36.36
Nursing	3	13.64
Community health	2	9.09
Unknown	4	18.18
Working years		
<5	9	40.91
5-10	6	27.27
>10	8	36.36
Maternal or Child healthcare worker*		
Maternal healthcare worker	6	28.57
Child healthcare worker	8	38.10
Maternal and child healthcare worker	7	33.33

*1 was missing for maternal or child healthcare worker variable.

### Coverage and service providers of postnatal care

Of 1601 caregivers enrolled, 1442 (90%) completed the questionnaire module on delivery and postnatal care. The reasons for not completing the module were that the caregiver was not the mother (158) or not familiar with postnatal care (1). More people from non-responder group were from urban areas, had higher education (high school and above), worked as staff and smaller family size (Additional file 4). Only 110 (8%) received a timely postnatal home visit (within 1 week after delivery) (Table 3). A total of 165 (13%) mothers reported that they had been visited during the postnatal period at home; over half of them were visited by village doctors (54%) followed by township doctors (38%). A higher proportion (24%) of mothers received postnatal care within 42 days after delivery; 50% of them went to hospitals where they delivered the baby and 44% of them went to township hospitals.

**Table 3:** **Postnatal care, providers and places in rural Hebei, China, 2011**^**$**^

**Postnatal care**	**n**	**%**	**Total**
Postnatal home visit			
Any	165	11.47	1439
Within 1 week	110	7.64	1439
Providers			162
Village doctors	87	53.71	
Township doctors	62	38.27	
Nurse	2	1.23	
MCH worker	7	4.32	
Midwife	2	1.23	
Family Planning staff	1	0.62	
Family/relatives	1	0.62	
Postnatal care within 42 days			
Any	342	24.07	1421
Place			338
Township hospital	147	43.49	
Delivery hospital	168	49.70	
Others	23	6.81	

^$^3 were missing for any postnatal home visit and postnatal home visit within 1 week respectively, 3 were missing for providers of postnatal home visit, 21 were missing for postnatal care within 42 days and 4 were missing for places of postnatal care within 42 days.

MCH: Maternal and child healthcare.

In our qualitative interview, we also found that township MCH workers and village doctors both conducted postnatal home visits. However, the work allocation and responsibility for MCH workers and village doctors were not defined or scheduled both at township and village level, and even not within township level. In some townships, township maternal and child healthcare workers visited homes. In other townships, village doctors and township maternal or child healthcare workers performed home visits. The different health workers visited homes separately or together.

I did half of the home visits myself and village doctors did the other half of the work. Sometimes, I even use telephone calls to check the newborns. (Child healthcare worker, township 13)

### Services provided during postnatal home visit

From our quantitative survey, among 164 mothers who were visited at home by health workers (township doctors, village doctors, nurses, MCH workers, midwives and family planning staff) postnatal, 40% of their newborns were weighted and guided on growth monitoring (Table 4). Only 37% of them received counseling on infant feeding and 32% on cord care. Only 24% of surveyed mothers said that their newborns were checked on jaundice. A very small proportion of mothers (18%) were consulted on the danger signs of newborns and keeping newborns warm. Except for measure weight and provide guidance, the proportion of services provided by village doctors during postnatal home visits (check jaundice, counseling/guidance on danger signs of newborn, feeding, cord care and keeping warm) were higher than MCH workers. However, none of the services differed significantly among various providers.

**Table 4:** **Quality of postnatal care among various providers in rural Hebei, China, 2011 (N = 164) **^**$**^

**Quality of Postnatal Care**	**Total**	**Township MCH doctors/workers**	**Village doctors**	Others*	P value**
		**(n = 62)**	**(n = 87)**	**(n = 13)**	
Measure weight and provide guidance	65(39.63)	31(50.00)	29(33.33)	4(30.77)	0.097
Check jaundice	40(24.39)	15(24.19)	23(26.44)	1(7.69)	0.337
Counseling/guidance					
Danger signs of newborn	29(17.68)	8(12.90)	19(21.84)	1(7.69)	0.231
Feeding	61(37.20)	19(30.65)	36(41.38)	5(38.46)	0.406
Cord care	53(32.32)	16(25.81)	31(35.63)	5(38.46)	0.393
Keep warm	29(17.68)	10(16.13)	17(19.54)	0(0.00)	0.209

^**$**^2 were missing for service provider.

*Others: nurse, midwife and family planning staff.

**Pearson chi-square test was used to compare difference among various providers.

MCH: Maternal and child healthcare.

In qualitative interviews, MCH workers also mentioned the following services during home visits: checking for jaundice, checking the cord, measuring the body temperature, length and weight for newborns and checking lochia and suture line of the mother and the general situation for “sitting the month”. Only one maternal healthcare worker said to follow national guidelines during postnatal home visits:

I checked the pregnant women by following the items required from maternal and child healthcare booklet one by one. (Maternal healthcare worker, township 17)

None of interviewed MCH workers mentioned to provide infant feeding counseling and guidance on postnatal danger signs of the mother and child.

### Willingness and reasons for not receiving the services

From our survey, most (91%) mothers expressed their willingness to be visited by health workers after delivery. A very small proportion (2.5%) of mothers reported they did not want visitors during the postnatal period.

Women who sought care within 42 days after delivery did not differ significantly to women who did not seek care in terms of maternal age, Hukou, education, occupation, family size, annual family income, annual family expenditure and children’s gender (Table 5).

**Table 5:** **Comparison between women who did and did not seek care within 42 days after delivery in rural Hebei, China 2011(n = 1421)**^**$**^

	**Women sought care**	**Women who did not seek care**	**P value**
	**(n = 342)**	**(n = 1079)**	
Maternal age, yr*	27(24–30)	27(25–30)	0.436
Maternal Hukou**			1.000
Urban	3(0.88)	11(1.03)	
Rural	336(99.12)	1058(98.97)	
Maternal education***			0.762
Primary school	15(4.45)	56(5.29)	
Junior high school	269(79.82)	857(81.00)	
High school	44(13.06)	119(11.25)	
College or above	9(2.67)	26(2.46)	
Maternal occupation***			1.000
Farmer	257(75.82)	812(75.96)	
Worker/Staff	7(2.06)	22(2.06)	
Housewife	64(18.88)	201(18.80)	
Others	11(3.24)	34(3.18)	
Family size*	5(4–6)	5(4–6)	0.949
Annual family income, Yuan*	20000(10000–30000)	20000(10000–30000)	0.065
Annual family consumption expenditure, Yuan*	15000(10000–20000)	15000(10000–20000)	0.609
Children’s gender***			0.959
Male	198(57.89)	623(57.74)	
Female	144(42.11)	456(42.26)	

^$^Continuous variables were expressed as median (Q1-Q3) and categorical variables were expressed as number (percentage).

*Two-sample wilcoxon rank-sum test was used to compare continuous variables between women who did and did not seek care within 42 days after delivery.

**Fisher exact test was used compare categorical variables between women who did and did not seek care within 42 days after delivery.

***Pearson chi-square test was used compare categorical variables between women who did and did not seek care within 42 days after delivery.

**Figure 1:**
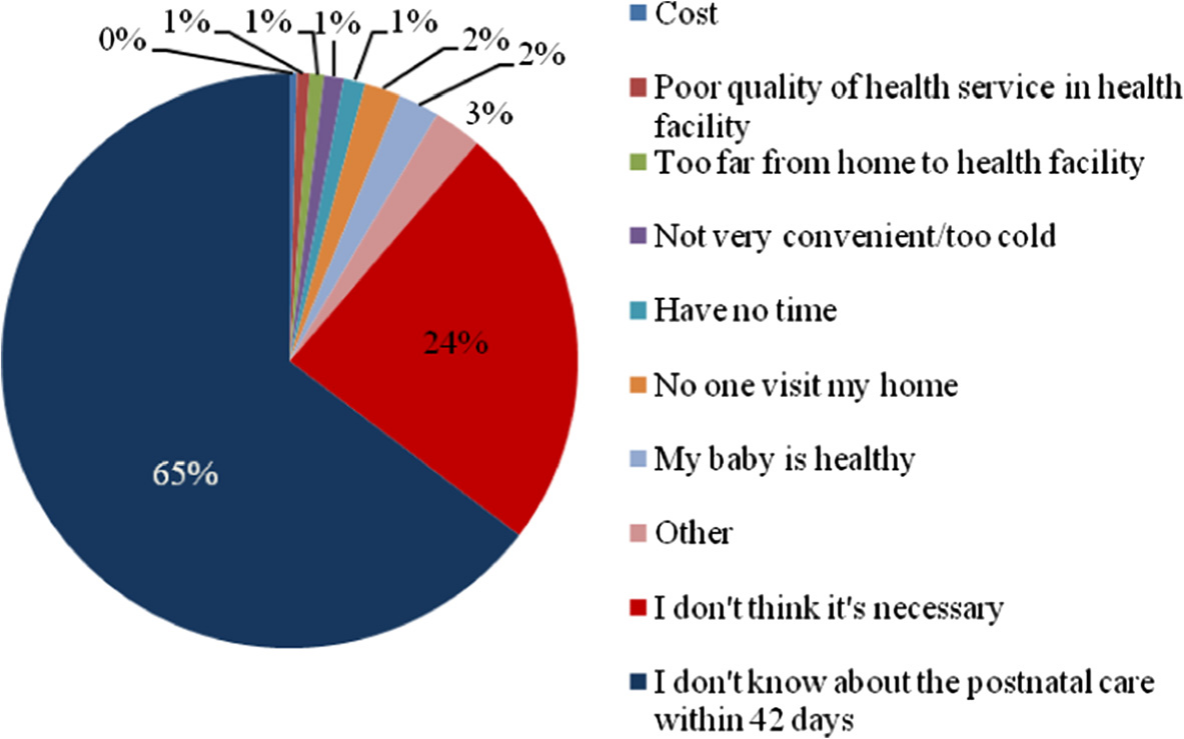
Reasons for not having postnatal care within 42 days after delivery from mothers in rural Hebei, China 2011.

Of 991 mothers who did not seek postnatal care within 42 days after birth, 65% of them said that they did not know about postnatal care and 24% of them thought it was unnecessary (Figure 1). However, very few (4%) mothers mentioned accessibility factors (such as cost, and too long distance from home to health facility) as barriers for them to seek postnatal care services.

### Barriers for MCH workers to conduct postnatal home visits

Regarding the barriers to postnatal home visits, we found three subthemes from qualitative interviews: 1) understaffing, 2) inadequate in-service training, and 3) inconvenient transportation.

Understaffing restricted MCH workers to conduct postnatal home visits. MCH workers sometimes needed to work extra hours, either early in the morning or after work in the evening, to visit women at their homes. Also, because MCH workers undertook other work such as antenatal care, well-baby clinic visits and filling in personal health record forms, they found it difficult to deal with the various work requirements. Therefore, they had no time to reach out to women at home. To improve coverage of postnatal care, MCH workers suggested increasing the number of MCH workers who conduct home visits or establishing a collaboration mechanism with village doctors.

“[It is] better to have two child healthcare workers [in each township].” (Child healthcare worker, township 2)

“[We need to] collaborate with village doctors. Communicate with them well and then conduct work jointly with village doctors.” (Maternal healthcare worker, township 17)

In some villages, there were no female village doctors and MCH workers reported that it was culturally inappropriate for male village doctors to visit postnatal women at their home.

“The village doctors in some villages are not female. Generally, when a woman is sitting month at home, it’s not comfortable to have a male to visit.” (Child healthcare worker, township 13)

After the initiation of national public health services, township MCH workers were trained for seven days at Shijiazhuang, the capital city of Hebei Province. MCH workers also received in-service training at a county maternal and child healthcare hospital. MCH workers described that training included a wide range of topics: introducing guidelines of national basic public health services, maternal healthcare, child healthcare, communication skills, breastfeeding and complementary feeding, personal health records, child growth and monitoring and folic acid supplementation. However, the training was mainly focused on how to fill in various forms such as personal health records or theoretical lectures. Postnatal care was not mentioned as a topic that was specifically covered in these in-service trainings.

“I was trained at the county maternal and child healthcare hospital… [The training] was about how to fill out the forms.” (Maternal and child healthcare worker, township 12)

“The training was in this July and they gave us lectures… [They talked about] basic public health services and personal health record. [They taught us] what items are in the personal health record and how to fill out the record form.” (Maternal and child healthcare worker, township 18)

In-service training was seen as inadequate to meet the needs of MCH workers to provide postnatal care of adequate quality. The majority of interviewed MCH workers expressed that they wanted more trainings in terms of neonatal diseases, common gynecological diseases, how to care for women during the postnatal period, and how to manage breastfeeding problems such as nipple cracks. Regarding the format of training, MCH workers expected to gain more practical experience during the training and to receive more supervision after training. Also, MCH workers suggested training village doctors to improve their skills.

“I hope the teacher could walk us through the [healthcare service] process, so they could tell us how to manage if something happens and I can also ask [while practicing] if I have questions.” (Maternal and child healthcare worker, township 8)

MCH workers mentioned that inconvenient transportation also limited them in performing postnatal home visits. Health facilities did not provide vehicles for MCH workers to conduct postnatal home visits and the public transportation at village level was not convenient. Township MCH workers often rode their own bike, electric bike or motorcycle to reach out to women and their newborns at home in the postnatal period. The poor road conditions, long travel distances and dispersed distribution of villages within a township also negatively affected the ability of MCH workers to visit women at home. Especially heavy rain worsened the road conditions and made it impossible for MCH workers to travel to villages.

For me, it took usually half an hour or an hour to travel from my office to village before I started to visit women at home. And the transportation itself is not convenient. The time schedule is very tight to fulfill the requirement of postnatal home visit. (Maternal and child healthcare worker, township 11)

## Discussion

Our study found that after initiation of free postnatal care in rural Hebei Province, only 8% of mothers received a timely postnatal home visit and one in four received postnatal care within 42 days after delivery. The coverage estimated in our study was lower than the results from the NHSS survey in 2008, in which 54% of women in rural areas received at least one postnatal visit. However, the definition of a postnatal visit in NHSS neither specified the timing of care nor differentiated care from a home visit, or phone call or health facility visit [21]. The coverage in our study, though slightly higher, was more in line with results from a study in rural Anhui Province in 2005–2006, which reported coverage of 4.2% in one county and 4.5% in a second county [22].

Neonatal mortality in rural areas was higher than in urban areas in China [6] and an effective community-based intervention package to improve neonatal survival has been established [13]. Promotion of early breastfeeding, thermal care, cord care and education on danger signs of care seeking together with clean delivery and home management of low birth weight infants can reduce neonatal mortality with 20-30% [13]. For the small proportion of women who received postnatal care, the quality of care was poor. The guideline of postnatal home visit was issued [30], but in our study the guideline was poorly followed by township level MCH workers. Both our quantitative and qualitative results identified that neonatal physical checks on weight and jaundice and counseling on feeding, cord care, thermal care and danger sighs were suboptimal. Similar findings also found from another study in Fujian Province, China interviewed 776 women after delivery on services content they received during postnatal home visit and very few reported to have checked on neonatal health [42].

The low coverage and poor quality of postnatal care reflects a continued neglected component of maternity services and a gap in the continuum of care. We identified barriers from both the supply and demand side. Demand for care is determined by whether an individual identifies the needs for care and is able to seek appropriate care [43]. Demand barriers identified from previous studies were mainly related to the traditional Chinese “sitting month” phenomenon that restricts women from outdoor activities and visits from people during the first month after delivery [22,34]. Therefore, the demand for home visits after birth perceived by health workers is inadequate [35]. This may not only limit women in receiving adequate care, but also demoralize health workers to provide services [44]. However, our study found that over 90% of women were willing to be visited by health workers during the postnatal period. A qualitative study from Fujian Province, China also revealed that mothers seemed to be happy to receive visitor and to have more social interaction. This finding may suggest that the attitude of mothers towards the “sitting month” custom of restricting visitors has changed [22]. However, a health worker in our study said that women still reported that they may not be comfortable with visits from male doctors during the “sitting month” period.

We also found the major reasons for not seeking care within 42 days after delivery from the demand side were that mothers did not know about postnatal care and thought it was unnecessary. The national initiative provided free service to all women and newborn after delivery to reduce the barriers to affordability. However, this does not address the information barrier on the demand side. Most women (99.6%) deliver at hospitals in our survey (data not shown) and this can be used to educate women about the timing and schedule of home visit one week after birth and health check at health facility within 42 days before discharging them. In Malawi, an education initiative at a delivery hospital reported to increase the use of hospitals and clinics for postnatal care [45]. A recent study showed that behavior change communication during antenatal care can promote demand for skilled intrapartum and postnatal care, indicating that awareness thus could be raised by a notification in advance [46].

Regarding the supply side, shortages of staff, inadequate in-service training and inconvenient transportation limited township level MCH workers in providing postnatal home visits of adequate quality. Previous studies found that understaffing and inadequate funding at township and village level health facilities are the main barriers from the supply side [22]. Township or village health facilities were a solid foundation of China’s three-tier maternal and child health networks in 1960s and 1970s [47]. However, from late 1970s, village clinics were privatized and three-tier maternal and child healthcare networks weakened after the economic reform [30]. In 2009, the national basic public health policy initiated and postnatal care was identified as one of the free public health services to all women in both rural and urban areas [34]. The Chinese government has allocated earmarked funding for maternal and child health and enhanced infrastructure and in-service training [48]. However, they did not specify how work should be allocated at township and village level and how staff shortages should be dealt with [29]. Recruiting more MCH staffs at township level may not be an immediate option when there is limited healthcare funding. Therefore, a more feasible approach may be to focus on streamlining the work arrangements and establishing a collaboration mechanism between township level MCH workers and village doctors. Village doctors reside within rural community and are familiar with women and their families. Moreover, the relationship between village clinics and township health facilities are important for improving public health services provided by village doctors [28]. Therefore, it may geographically be more feasible and more acceptable for village doctors to conduct postnatal home visits in their own communities. And the transportation barriers encountered by township level MCH workers can also be resolved by relying on village doctors to visit women and children at home during postnatal period. Township MCH workers on the other hand might act as supervisors to monitor and feedback on the performance of village doctors and to bridge care between the family, community and health facility.

In-service training was found to be inadequate, which constituted to another obstacle in improving the coverage and quality of postnatal care. Our study found that current in-service training was mainly focused on paper work, rather than on improving the coverage and quality of healthcare service. Also, township level MCH workers expressed their desire for more hands-on practice and supervision. Therefore, a specific training session on postnatal care is needed and this may include the identification and selection of service providers, assessing the knowledge of their function and needs, and provision of job descriptions [49]. Supervision by structured observations and standard checklists, meetings with staff, self-assessment, demonstration and provision of feedback has been shown to improve the skills of health professionals [50].

Our study provided important preliminary insights in how postnatal care was provide in a setting in rural China after the national initiation of free postnatal care in 2009. The use of both quantitative and qualitative techniques improved our understanding of MCH worker’s views on barriers toward postnatal care services. However, our study also has some limitations. Firstly, we did not interview village doctors to assess their training, workload, perceptions about postnatal care work, while we found that village doctors carried out half of the postnatal home visits in their catchment areas. Further research on exploring perceptions and experiences of village doctors in providing postnatal care is needed. Secondly, the name list used in our study may not be complete. In Zhao County, all live births are reported to the county level Maternal and Child Health Hospital from all qualified delivery institutions each month. We obtained the name list from the hospital and then sent the name lists of all selected villages to village doctors and asked them to remove children who had died or moved outside the village, and we added children who were living in the village but who were not on the list. Although all these efforts were made, we were not sure about the completeness of the name lists. Besides, despite our high response rate, non-respondents compared to respondents of our household survey differed in some general characteristics (maternal Hukou, education, occupation and family size). A higher proportion of non-respondents had an urban maternal Hukou, attended high school, college or above, were worker or staff, and had ≤3 persons per family. Therefore, selection bias may have influenced the generalizability of our study. Thirdly, we conducted interviews after interviewers observed the participants performing care and the interviewers checked MCH workers’ performance (this was part of the trial). The participants may not have told us what they really thought or they may have been reluctant to elaborate, because they may have been afraid of the interviewer’s judgment on their work performance. However, interviewers emphasized at the beginning of interview that the aim of the interview was not to judge their work performance and that the results were anonymous and confidential. Lastly, the quality of postnatal care in our study only included services for neonatal home visits. Therefore, our study may not provide a comprehensive review on all aspects of quality of postnatal care. However, no consistent postnatal care indicators in household survey globally have been established to measure the content or quality of postnatal care [51]. Our study provides insight in raising awareness of strengthening the quality besides from increasing coverage of postnatal care.

When addressing the barriers to postnatal care, a collaboration mechanism that links township-level MCH workers and village doctors should be established to ensure adequate staffing and to overcome geographical barriers for MCH workers to provide services. Additional in-service training on postnatal care is required for both township-level MCH workers and village doctors. Health workers should inform women about free postnatal care services and tell them about its importance. In addition, continuous monitoring and evaluation will further strengthen the skills of service providers, which in turn can improve the quality of postnatal care. Postnatal care in a health facility within 42 days after delivery also needs to be improved substantially. A more thorough study from both the demand side and supply side is needed to explore efforts that can improve facility services.

## Conclusion

Coverage and quality of postnatal care was low in rural Hebei, China. Our study identified both supply side and demand side barriers to postnatal care visits. Among mothers, there was a lack of awareness on the availability of free postnatal services among women, but also a strong willingness for receiving postnatal care. Staff shortages, inadequate in-service training and inconvenient transportation limited township level MCH workers in providing postnatal home visits of adequate quality. More village doctors need to be involved, better trained, so that they are able to provide postnatal home visits of adequate quality. Also, women should be informed about freely available postnatal care and its importance.

## Competing interests

The authors declare that they have no competing interests.

## Authors’ contributions

The study was initiated by ZT and ZYF. ZYF, ZSY, WQ, LY, WW and DXZ collected and coded the data. CL performed quantitative data analysis. CL and WQ performed the qualitative analysis with MV’s technical support. ZT and ZYF supervised the study and participated in the explanation and discussion of the results. The manuscript was drafted by CL, reviewed, and substantially revised by ZT, ZYF, WQ, LY, WW, DXZ and MV. All authors read and approved the final manuscript.

## Pre-publication history

The pre-publication history for this paper can be accessed here:

http://www.biomedcentral.com/1471-2393/14/31/prepub

## Supplementary Material

Additional file 1: Table S1 Timing, provider and postnatal services for mother and child according the national norm in China in 2010. Click here for file

Additional file 2 Sample size calculation. Click here for file

Additional file 3 Interview topic guide for interviewing maternal and child healthcare workers at township level, Zhao County, Hebei Province, China. Click here for file

Additional file 4: Table S2 Comparison of general characteristics between respondents and non-respondents. Click here for file

## References

[B1] World Health Organization WHO Technical Consultation on Postpartum and Postnatal Care 2010 Geneva, Switzerland: WHO Document Production Services 26269861

[B2] MacArthur C Winter HR Bick DE Knowles H Lilford R Henderson C Lancashire RJ Braunholtz DA Gee H Effects of redesigned community postnatal care on womens’ health 4 months after birth: a cluster randomised controlled trial Lancet 2002 359 9304 378 385 10.1016/S0140-6736(02)07596-7 11844507

[B3] The UN Inter-agency Group for Child Mortality Estimation Levels and trends in child mortality: report 2013 2013 New York, US: UNICEF

[B4] Chopra M Campbell H Rudan I Understanding the determinants of the complex interplay between cost-effectiveness and equitable impact in maternal and child mortality reduction J Glob Health 2012 2 1 10406 23198135 10.7189/jogh.02.010406 PMC3484756

[B5] Feng XL Guo S Hipgrave D Zhu J Zhang L Song L Yang Q Guo Y Ronsmans C China’s facility-based birth strategy and neonatal mortality: a population-based epidemiological study Lancet 2011 378 9801 1493 1500 10.1016/S0140-6736(11)61096-9 21924764

[B6] Feng XL Theodoratou E Liu L Chan KY Hipgrave D Scherpbier R Brixi H Guo S Chunmei W Chopra M Black RE Campbell H Rudan I Guo Y Social, economic, political and health system and program determinants of child mortality reduction in China between 1990 and 2006: a systematic analysis J Glob Health 2012 2 1 10405 23198134 10.7189/jogh.02.010405 PMC3484751

[B7] Ministry of Health of People’s Republic of China Summary Report Of Health Statistics In China, 2012 http://www.moh.gov.cn/zwgkzt/ptjty/201206/55044.shtml

[B8] Bick DE MacArthur C The extent, severity and effect of health problems after childbirth British Journal of Midwifery 1995 3 1 27 31

[B9] Glazener C Abdalla M Stroud P Templeton A Russell IT Naji S Postnatal maternal morbidity: extent, causes, prevention and treatment BJOG 1995 102 4 282 287 10.1111/j.1471-0528.1995.tb09132.x 7612509

[B10] Mao XM Sun XF Liu LG Hao LP Yao P Yang XF Study on maternal morbidity during the puerperium and its relation to dietary and behavior practice Chin J of Maternal and Child Health 2006 21 8 1038 1040

[B11] MacArthur C Winter HR BickDE LRJ LancashireRJ KH Braunholtz DA Henderson C Belfield C Gee H Redesigning postnatal care: a randomised controlled trial of protocol-based midwifery-led care focused on individual women’s physical and psychological health needs Health Technol Assess 2003 7 37 10.3310/hta7370 14622490

[B12] Qian YR Yan XY Prevmence of postpartum depression in China: a systematic analysis Chin J PracNurs 2013 29 12 1 3

[B13] Darmstadt GL Bhutta ZA Cousens S Adam T Walker N de Bernis L Lancet Neonatal Survival Steering Team Evidence-based, cost-effective interventions: how many newborn babies can we save? Lancet 2005 365 9463 977 988 10.1016/S0140-6736(05)71088-6 15767001

[B14] Xiong W He JY Xiao JC Early psychological intervention to improve maternal postpartum depression Chin Jof Nervous and Mental Diseases 2006 32 2 149,178

[B15] Wang SL Preliminary study on development a puerperal maternal family care model Prac J of Medicine 2006 22 14 1694 1695

[B16] Dhakal S Chapman GN Simkhada PP van Teijlingen ER Stephens J Raja AE Utilisation of postnatal care among rural women in Nepal BMC Pregnancy Childbirth 2007 7 19 10.1186/1471-2393-7-19 17767710 PMC2075509

[B17] Regassa N Antenatal and postnatal care service utilization in southern Ethiopia: a population-based study Afr Health Sci 2011 11 3 390 397 22275929 PMC3260999

[B18] Jat TR Ng N San Sebastian M Factors affecting the use of maternal health services in Madhya Pradesh state of India: a multilevel analysis Int J Equity Health 2011 10 1 59 9276-10-59 10.1186/1475-9276-10-59 22142036 PMC3283453

[B19] Moran Allisyn C Kerber K Sitrin D Guenther Y Morrissey CS Newby H Fishel J Yoder PS Hill Z Lawn JE Measuring coverage in MNCH: indicators for global tracking of newborn care PLoS medicine 2013 10 5 e1001415 10.1371/journal.pmed.1001415 23667335 PMC3646209

[B20] Ministry of Health China The management measure of systematic maternal healthcare in rural areas 1989 Beijing: Ministry of Health

[B21] Centre for Health Statistics and Information of Ministry of Health of People’s Republic of China An Analysis report of National Health Services Survey in China, 2008 2009 Beijing: Beijing Union Medical University Press

[B22] Tao F Huang K Long X Tolhurst R Raven J Low postnatal care rates in two rural counties in Anhui Province, China: perceptions of key stakeholders Midwifery 2011 27 5 707 715 10.1016/j.midw.2009.10.001 20850212

[B23] Koblinsky M Matthews Z Hussein J Mavalankar D Mridha MK Anwar I Achadi E Adjei S Padmanabhan P Marchal B De Brouwere V van Lerberghe W Lancet Maternal Survival Series steering group Going to scale with professional skilled care Lancet 2006 368 9544 1377 1386 10.1016/S0140-6736(06)69382-3 17046470

[B24] National Health and Family Planning Commission of the People’s Republic of China Primary responsibilities of Maternal and Child Health Services Divisionhttp://www.moh.gov.cn/fys/pzyzz/lm.shtml

[B25] Hesketh T Zhu WX Maternal and child health in China BMJ 1997 314 7098 1898 10.1136/bmj.314.7098.1898 9224139 PMC2126969

[B26] Chen L Dai Y Zhang Y Wu Q Rudan D Saftić V van Velthoven MH Su J Tan Z Scherpbier RW A comparison between antenatal care quality in public and private sector in rural Hebei, China Croat Med J 2013 54 2 146 156 10.3325/cmj.2013.54.146 23630142 PMC3641873

[B27] Shi LY Hung LM Song KM Rane S Tsai J Sun XJ Li H Meng QY Chinese primary care physicians and work attitudes Int J Health Serv 2013 43 2 167 181 23527460 10.2190/HS.43.1.k

[B28] Ding Y Smith HJ Fei Y Xu B Nie S Yan W Diwan VK Sauerborn R Dong H Factors influencing the provision of public health services by village doctors in Hubei and Jiangxi provinces, China Bull World Health Organ 2013 91 1 64 69 10.2471/BLT.12.109447 23397352 PMC3537250

[B29] Ministry of Health of People’s Republic of China Ministry Of Health Issued The Guidelines Of National Basic Public Health Services (2011 Version) http://www.gov.cn/zwgk/2011-05/24/content_1870181.htm

[B30] Liu Y Reforming China’s health care: for the people, by the people? Lancet 2009 373 9660 281 283 10.1016/S0140-6736(09)60080-5 19167561 PMC7135703

[B31] Ministry of Health of People’s Republic of China Norm Of National Basic Public Health Services 2009 http://www.gov.cn/jrzg/2009-07/10/content_1362010.htm

[B32] Ministry of Health of People’s Republic of China Central Government Allocated 10.4 Billion Yuan For Basic Public Health Serviceshttp://www.gov.cn/gzdt/2009-07/06/content_1358394.htm

[B33] Ministry of Health of People’s Republic of China News Release From Ministry Of Health On Introducing Health Reformfor Health System In Chinahttp://www.gov.cn/xwfb/2009-04/10/content_1282161.htm

[B34] Liu N Mao L Sun X Liu L Chen B Ding Q Postpartum practices of puerperal women and their influencing factors in three regions of Hubei, China BMC Public Health 2006 6 274 10.1186/1471-2458-6-274 17087836 PMC1636040

[B35] Raven JH Chen Q Tolhurst RJ Garner P Traditional beliefs and practices in the postpartum period in Fujian Province, China: a qualitative study BMC Pregnancy Childbirth 2007 7 8 10.1186/1471-2393-7-8 17584930 PMC1913060

[B36] Hebei Provincial Bureau of Statistics Statistical Communiqué Of The Hebei Province On Economic And Social Development Of 2010 2010 Shijiazhuang: Hebei Provincial Bureau of Statistics

[B37] National Bureau of Statistics of China Statistical Communiqué of the People’s Republic of China on National Economic and Social Development of 2010 2010 Beijing: National Bureau of Statistics of China

[B38] Ostlund U Kidd L Wengström Y Rowa-Dewar N Combining qualitative and quantitative research within mixed method research designs: a methodological review Int J Nurs Stud 2011 48 3 369 383 10.1016/j.ijnurstu.2010.10.005 21084086 PMC7094322

[B39] Sandelowski M Combining qualitative and quantitative sampling, data collection, and analysis techniques in mixed-method studies ResNurs Health 2000 23 3 246 255 10.1002/1098-240x(200006)23:3<246::aid-nur9>3.0.co;2-h 10871540

[B40] Zhang S Wu Q van Velthoven MH Chen L Car J Rudan I Zhang Y Li Y Scherpbier RW Smartphone versus pen-and-paper data collection of infant feeding practices in rural China J Med Internet Res 2012 14 5 e119 10.2196/jmir.2183 22989894 PMC3510690

[B41] Ritchie J, Lewis J Qualitative research practice: A guide for social science students and researchers 2003: Sage 219 262

[B42] Chen QY Chen LP Zhang RL Ye BF Feng YQ Zhuo XY Research on the quality of community postpartum visits in Fujian Chin J of Maternal and Child Health 2005 20 5 534 535

[B43] Ensor T Cooper S Overcoming barriers to health service access: influencing the demand side Health Policy Plan 2004 19 2 69 79 10.1093/heapol/czh009 14982885

[B44] Mrisho M Obrist B Schellenberg JA Haws RA Mushi AK Mshinda H Tanner M Schellenberg D The use of antenatal and postnatal care: perspectives and experiences of women and health care providers in rural southern Tanzania BMC Pregnancy Childbirth 2009 9 10 2393-9-10 10.1186/1471-2393-9-10 19261181 PMC2664785

[B45] Gennaro S Thyangathyanga D Kershbaumer R Thompson J Health promotion and risk reduction in Malawi, Africa, village women J ObstetGynecol Neonatal Nurs 2001 30 2 224 230 10.1111/j.1552-6909.2001.tb01539.x 11308113

[B46] Bhutta ZA Darmstadt GL Hasan BS Haws RA Community-based interventions for improving perinatal and neonatal health outcomes in developing countries: a review of the evidence Pediatrics 2005 115 Suppl2 519 617 15866863 10.1542/peds.2004-1441

[B47] Zhang D Unschuld PU China’s barefoot doctor: past, present, and future Lancet 2008 372 9653 1865 1867 10.1016/S0140-6736(08)61355-0 18930539

[B48] Chen Z Launch of the health-care reform plan in China Lancet 2009 373 9672 1322 1324 10.1016/S0140-6736(09)60753-4 19376436

[B49] Oyediran MA The importance of training and supervision in quality of care AdvContracept 1993 9 2 175 178 10.1007/BF01990148 8237570

[B50] Ozek B Saat Z Temiz AT Kinzie B On-the-job training through follow-up visits to improve the quality of family planning services Eur J ContraceptReprod Health Care 1998 3 4 201 206 10.3109/13625189809167254 10036603

[B51] Warren C Mwangi A Oweya E Kamunya R Koskei N Safeguarding maternal and newborn health: improving the quality of postnatal care in Kenya Int J Qual Health Care 2010 22 1 24 30 10.1093/intqhc/mzp050 19946120

