# Supplementary material for: Coverage, quality of and barriers to postnatal care in rural Hebei, China: a mixed method study
Source: BMC Pregnancy Childbirth. 2014 Jan 18;14:31. doi: 10.1186/1471-2393-14-31 (PMC3898028; doi:10.1186/1471-2393-14-31)
Supplement: Additional file 1: Table S1 — Timing, provider and postnatal services for mother and child according the national norm in China in 2010. [file 1471-2393-14-31-S1.doc]

**Additional file 1: Table S1 Timing, provider and postnatal services for mother and child according the national norm in China in 2010^1^**

| **Timing** | **Provider** | **Services for the mother** | **Services for the child** |
| --- | --- | --- | --- |
| Postnatal home visit 3-7 days after discharge from hospital | Urban area: Community health service centers (stations)  Rural area: Township hospitals and village clinics | Assess the general situation of maternal breast, uterus, lochia, perineum or abdominal wound healing and so on through observation, inquiry and examination.  Manage and consult on maternal postpartum care, on breastfeeding difficulties, postpartum constipation, hemorrhoids, and perineal or abdominal wounds.  Refer women to higher level health facilities if identified with puerperal infection, postpartum hemorrhage, poor uterine involution, and prolonged pregnancy complications and postpartum depression. | Ask about the situation at birth, immunization status and neonatal screening where neonatal screening has been implemented.  Observe the home environment.  Ask and observe feeding, sleeping, defecation, jaundice, umbilical cord, oral development and so on.  Measure and record body temperature, birth weight, current body length.  Conduct physical examination and establish of "0 to 6 years old child health handbook."  Consult and guide on breastfeeding, newborn care and common disease prevention.  If the newborn has not received BCG vaccination or a hepatitis B vaccine, remind parents to replant.  If the newborn has not received neonatal screening, advise parents to have the screening.  For low birth weight, premature birth, twins or triplets or children born with birth defects, increase number of visits. |
| Postnatal health facility care within 42 days after delivery | Healthy postpartum urban women go to community health service centers (stations) and healthy postpartum rural women go to township hospitals.  High risk postpartum women go back to their delivery hospital. | Conduct general physical examination and gynecological examination, perform additional tests to assess the situation of maternal recovery when necessary.  Consult on sexual health, contraception, prevention of reproductive tract infections, exclusive breastfeeding for six months, infant nutrition and so on. | Inquiry and observe children’s feeding, sleeping, defecation, jaundice and so on.  Measure and record weight, height and conduct physical examination and developmental assessment. |

[1] Ministry of Health of People’s Republic of China: **Ministry of Health issued the guidelines of national basic public health services (2011 version)** [<http://www.gov.cn/zwgk/2011-05/24/content_1870181.htm>]
