# Supplementary material for: Coverage, quality of and barriers to postnatal care in rural Hebei, China: a mixed method study
Source: BMC Pregnancy Childbirth. 2014 Jan 18;14:31. doi: 10.1186/1471-2393-14-31 (PMC3898028; doi:10.1186/1471-2393-14-31)
Supplement: Additional file 2 — Sample size calculation. [file 1471-2393-14-31-S2.doc]

Sample size calculation

*Rationale:*

The data in this paper were generated as part of a broader research project on maternal and child healthcare services in rural China, Zhao County in Hebei Province, entitled ‘Effectiveness of a scaling-up model for child health interventions: a cluster randomized control trial’ (unpublished). The sample size and sampling method for the quantitative household survey was based on the cluster randomized control trial in Zhao County and the survey was used as the baseline assessment for the trial. We used townships as the randomization unit (8 townships per group). One of the hypotheses of this trial was that infant feeding counseling systematically delivered through multiple delivery channels improves nutritional status significantly better than routinely delivered feeding counseling.

*Sample size calculation:*

We expected to achieve a 10% point reduction of anemia prevalence in infants, and at least a 20% point increase inknowledge and practice of appropriate feeding for caregivers. With 80% power and a 5% significance level, we calculated that the sample size of 800 children under the age of two per group would be sufficient for all key indicators. We over-sampled 30% to compensate for possible refusal and loss to follow-up. Younger children were preferentially sampled to ensure a sufficiently large sample size in the younger age groups and to capture all relevant indicators for infant and young child feeding.

*Sampling:*

We used a two-stage sampling procedure. In the first stage, 160 clusters (villages) were selected using proportional to population size sampling, with 10 clusters chosen from each township. In the second stage, the name list of all eligible children under two years in each village was obtained and children were randomly selected using computer generated random numbers.
