# Supplementary material for: Coverage, quality of and barriers to postnatal care in rural Hebei, China: a mixed method study
Source: BMC Pregnancy Childbirth. 2014 Jan 18;14:31. doi: 10.1186/1471-2393-14-31 (PMC3898028; doi:10.1186/1471-2393-14-31)
Supplement: Additional file 3 — Interview topic guide for interviewing maternal and child healthcare workers at township level, Zhao County, Hebei Province, China. [file 1471-2393-14-31-S3.doc]

**Interview topic guide**

**for interviewing maternal and child healthcare workers at township level, Zhao County, Hebei Province, China**

Semi-structured interviews are informal conversations conducted between interviewers and interviewees aiming to explore the research questions in depth. The questions in semi-structured interviews are open-ended, which means that there are no fixed answers. So interviewers should not read questions word by word, as usually is done in surveys, but listen to the interviewee’s answer and follow the answers up with probing questions to ask for details and reasons behind answers. Interviewers should familiarize themselves with the topic of conversation and questions in order to make the interview run smoothly.

**Part I General characteristics**

| **Name of the institution** |  | | | | |
| --- | --- | --- | --- | --- | --- |
| **Interviewers** | No. | Name | | | |
| **Interview date** | (YYYY/MM/DD) | | | | |
| **Maternal and child healthcare worker** | No. | | Name |  | Age Yr |
|  | Gender | | 1 Male  2 Female | | |
|  | Administrative role | | 1 Dean  2 Associate dean  3 Others  4 No administrative role | | |
|  | Qualification | | 1 Certified physician  2 Certified assistant physician  3 None | | |
|  | Prescription privilege | | 1 Yes  2 No | | |
|  | Academic title | | 1 Junior level  2 Intermediate level  3 Vice-senior level  4 Senior level | | |
|  | Basic education | | 1 Primary school  2 Junior high school  3 Senior high School | | |
|  | Years of basic education | | 1 One year  2 2 years  3 3 years  4 4 years  5 5 years  6 6 years | | |
|  | Professional education | | 1 Vocational high school  2 Technical secondary school  3 Junior college diploma  4 College or above | | |

**Part II Basic public health services (include antenatal care, postnatal care, immunization and child health check)**

1 Medical Education

Q1 Have you received any formal medical education that is recognized by our nation? If so, what level? Start and end dates? The major?

2 Working experience

Q2 Do you now engage in any women and/or child healthcare work?

Q3 Do you have other responsibilities except for women and/or child healthcare services at work? If so, what are they?

Q4 How many years have you been working for women and/or child healthcare?

Q5 What did you do before you work on women and/or child healthcare?

Q6 How many women and/or children you see every day?

Q7 What do you think of your current workload?

Q8 What is the main work on which you spend most of your time?

Q9 Is there any work that you think is duplicated? Or do you think is unnecessary? Or you do not like to do?

Q10 Do you think pregnant women or children understand the basic public health services?

Q11 What’s your estimation of the proportion of women and/or child who have received basic public health services?

Q12 What measures have you taken to improve public’s understanding of basic public health services?

Q13 How to increase the awareness of public health services among general people?

3. Training

Q14 Have you received any training before you conducted basic public health services? If so, what was the content of that training? How long was the training? Did you pass the training?

Q15 Have you received any on-services training during the past two years?

Q16 Do you think that trainings was helpful? If so, in which way?

Q17 Among the basic public health services, are there any services that you are not familiar with that require further training?

Q18 Do you have any expectations on the content and format of training?

4. Postnatal home visit

Q19 Have you ever conducted postnatal home visits?

Q20 Do you think that it is necessary to conduct postnatal home visits?

Q21 How do you conduct postnatal home visits?

Q22 What do you think are the barriers to conduct postnatal home visits?

Q23 Do you need more training in order to better conduct postnatal home visits? If so, what training do you need specifically?

Q24 Do you have enough time to conduct every postnatal home visit in your catchment area?

Q25 How do you think the postnatal home visit can be improved?
