# Supplementary material for: Coverage, quality of and barriers to postnatal care in rural Hebei, China: a mixed method study
Source: BMC Pregnancy Childbirth. 2014 Jan 18;14:31. doi: 10.1186/1471-2393-14-31 (PMC3898028; doi:10.1186/1471-2393-14-31)
Supplement: Additional file 4: Table S2 — Comparison of general characteristics between respondents and non-respondents. [file 1471-2393-14-31-S4.doc]

Additional file 4: Table S2 Comparison of general characteristics between respondents and non-respondents*

|  | Respondents  (n=1442) | Non-respondents  (n=159) | P value |
| --- | --- | --- | --- |
| Maternal age, yr**** | 27(24-30) | 26(25-29) | 0.232 |
| Maternal Hukou** |  |  | 0.009 |
| Urban | 14(0.98) | 6(3.90) |  |
| Rural | 1415(99.02) | 148(96.10) |  |
| Maternal education** |  |  |  |
| Primary school | 74(5.23) | 8(5.67) | <0.001 |
| Junior high school | 1141(80.58) | 97(68.79) |  |
| High school | 166(11.72) | 21(14.89) |  |
| College or above | 35(2.47) | 15(10.64) |  |
| Maternal occupation*** |  |  | <0.001 |
| Farmer | 1083(75.79) | 103(66.88) |  |
| Worker/Staff | 30(2.10) | 34(22.08) |  |
| Housewife | 269(18.82) | 9(5.84) |  |
| Others | 47(3.29) | 8(5.19) |  |
| Family size*** |  |  |  |
| ≤3 persons per family | 172(11.93) | 29(18.24) | 0.023 |
| >3 persons per family | 1270(88.07) | 130(81.76) |  |
| Annual family income, Yuan^a****^ | 20000(10000-30000) | 20000(10000-30000) | 0.500 |
| Annual family consumption expenditure, Yuan^b****^ | 15000(10000-20000) | 15000(10000-20000) | 0.380 |
| Children's gender*** |  |  | 0.239 |
| Male | 832(57.70) | 84(52.83) |  |
| Female | 610(42.30) | 75(47.17) |  |

* Continuous variables were expressed as median (Q1-Q3) and categorical variables were expressed as number (percentage).

**Fisher exact test was used to compare categorical variables between respondents and non-respondents.

***Pearson chi-square test was used to compare categorical variables between respondents and non-respondents.

****Two-sample wilcoxon text was used to compare continuous variables between respondents and non-respondents.

a 549 were missing from respondents and 89 were missing for non-respondents.

b 520 were missing from respondents and 83 were missing for non-respondents.
